# A Bibliometric Analysis of Neuroinflammation in Depression from 2004 to 2023: Global Research Hotspots and Prospects

**DOI:** 10.7150/ijms.100888

**Published:** 2025-05-28

**Authors:** Anni Shi, Na Chen, Qin Ma, Yaxuan Wang, Xiaoling Liu, Jun Lu, Jianyou Guo

**Affiliations:** 1 College of Acupuncture-Moxibustion and Tui Na, Beijing University of Chinese Medicine, Beijing, China.; 2 The Second School of Clinical Medicine, Beijing University of Chinese Medicine, Beijing, China.; 3 Key Laboratory of Mental Health, Institute of Psychology, Chinese Academy of Sciences, Beijing, China.

**Keywords:** bibliometric analysis, depression, neuroinflammation, visualization analysis, research trends

## Abstract

**Background:** Neuroinflammation lays a prominent impact in the pathophysiology of depression, and numerous studies have been conducted in recent decades. Bibliometric analysis is of important for understanding the hot spots and research trends in a certain subject field. However, no systematic bibliometric study exists in this field to date. The purpose of the study focused on the trends and hotspots in neuroinflammation of depression and provided future researchers with guidance and sights.

**Methods:** Publications (2004-2023) were obtained from the WoSCC, and analyzed by HistCite, VOSviewer, CiteSpace, and Bibliometrix. The impact of publications was assessed by TGCS.

**Results:** We analyzed 1,496 articles published in 409 journals and authored by 46,533 researchers across 72 countries and regions. The most prolific countries were China, the USA, and Brazil, and the most cited countries were the USA, followed with China and the UK, while the most prolific and cited institution was University Toronto (records=34, TGCS=2,137). Brain Behavior and Immunity is the leading journal that regularly published research in this field (records=93, TGCS=6,247). NLRP3 inflammasome, microglia, TNF-α, and brain-derived neurotrophic factor (BDNF) were the basis of neuroinflammation in depression. C-reactive protein, an important marker of inflammation, has been discussed for the longest time in this disease. In recent five years, two most frontier potential areas in studying depression were gut microbiota dysbiosis and BDNF.

**Conclusions:** There remains a strong research basis for neuroinflammation in depression from this bibliometric analysis. Microglial activation, gut microbiota, cytokine signaling, and oxidative stress were research hotspots in recent years. In the future, chronic stress, hippocampal structure, and gut microbiota will continue to be studied in the field of neuroinflammation in depression. This study may benefit scientists in identifying potential directions for future study and providing clinicians with new ideas for treatment.

## Introduction

As a common mental illness, depression is featured with a marked and persistent low mood. Research data from the Annals of Clinical Psychology in 2022 suggested that depression case numbers have increased by nearly 50 percent occupation globally over the past 30 years 1. 350 million individuals worldwide suffered from depression spanning all age groups. Depression has been recognized as the third highest burden of disease globally and is expected to jump to the first place by 2030 2. In addition, depression is an important risk factor leading to disability according to the WHO, and about 1 million people commit suicide due to depression every year 3. Therefore, it is crucial to develop new approaches to treating and diagnosing depression.

As a complex mental illness, the development of depression is closely affected by numerous elements, such as genetic, psychological, biochemical, and socio-environmental factors 2,4. The main hypotheses about the pathogenesis of depression include the monoamine neurotransmitter hypothesis, hypothalamic-pituitary-adrenal (HPA) axis dysregulation hypothesis, neurotrophic factor hypothesis, and so on. Recent studies demonstrated that neuroinflammation has an influence on the onset and progress of depression 5,6. Neuroinflammation was characterized by activated microglia and released inflammatory cytokines in the central nervous system. Involved in the generation of cytokines from immune response process, microglia functioned in managing neurogenesis, synaptic interactions as well as neuronal cell death. It has been found that microglia activation occurred in a wide range of psychiatric disorders 7, which may be the target of antidepressant treatment for refractory depression 8. There was evidence that activated inflammatory pathways in the brain contributed to the reduction of neurotrophic support, the change of glutamate release or reuptake, and convergence of oxidative stress, which leads to the neuropathological changes of depression 9. The treatment of depression from the perspective of neuroinflammation deserves further study in the future.

Bibliometric analysis is an imperative statistical method for quantitatively analyzing large numbers of heterogeneous publications 10. Documents are analyzed multidimensionally using math, statistics, metrology, and other disciplines. Bibliometric analysis pays attention to the characteristics of literature, and it also provides reference and trends for future research direction in a specific field 11. During the past two decades, many publications have provided strong evidence for the relationship between neuroinflammation and depression 12-14, However, there is still a lack of systematic summaries of these studies. Therefore, this investigation intends to comprehensively review the current researches based on neuroinflammation in depression by employing bibliometric techniques, in order to explore the relationship between depression and neuroinflammation, and to find research hot topics and prevalent trends.

## Methods

### Data Source and Collection

**Figure 1:**
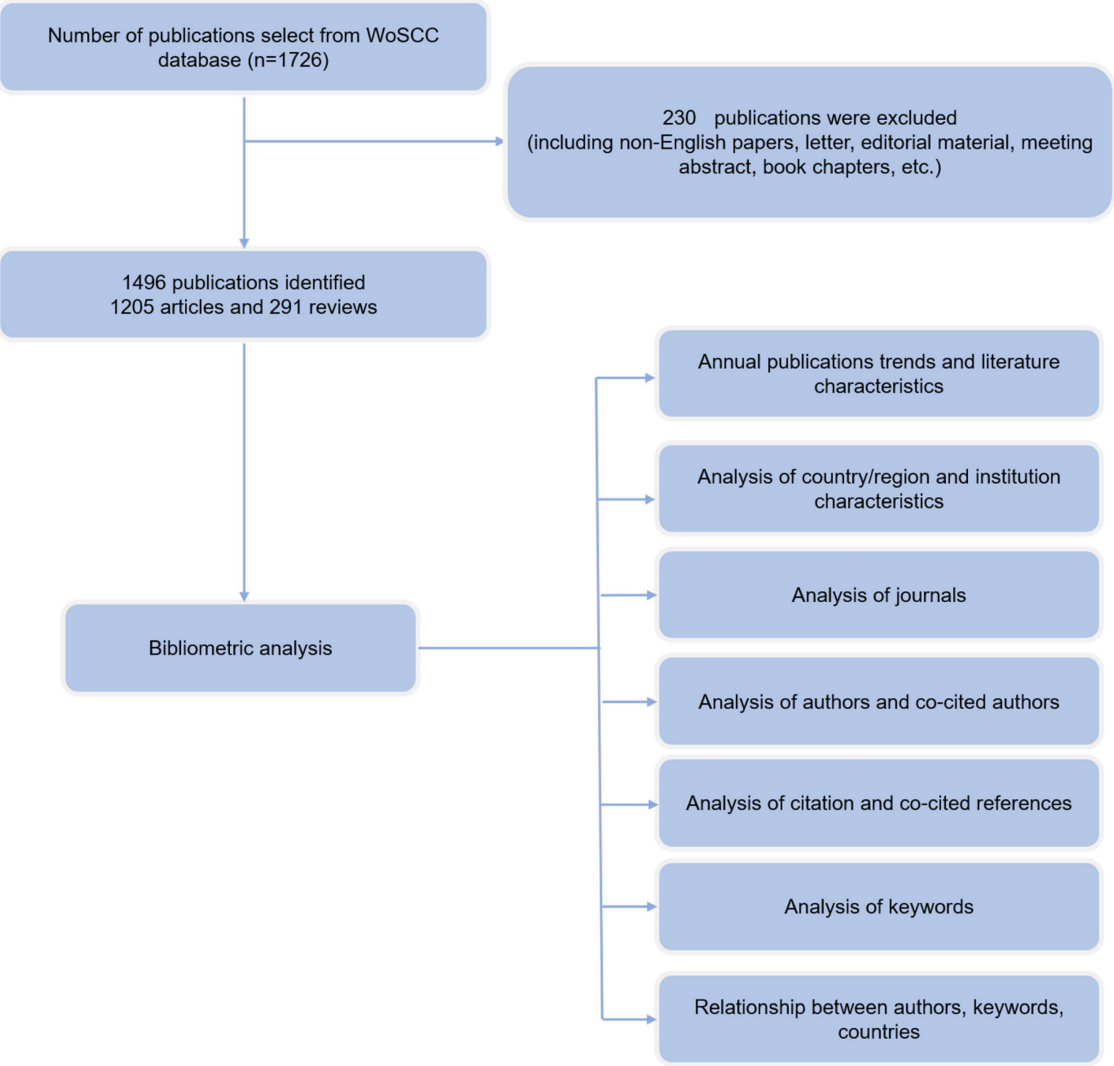
Inclusion and analysis flow chart.

Data was obtained from the Science Citation Index Expanded (SCI-Expanded) database of Web of Science (WoS) in this study. To acquire research on neuroinflammation in depression, we used the following search strategy to search for data from WoSCC: Title (“depressi*”) AND Topic (“Neuroinflammat*” OR “neurogenic inflammati*” OR “Nervous Inflammat*” OR “central inflammat*” OR “Neuroinflammation in the central nervous” OR “Brain inflammat*” OR “systemic inflammat*”) with a language restriction to English and limited time from January 1st, 2004 to October 1st, 2023. Only articles and reviews are permitted as forms of literature. Specifically, the operation process is described in **Figure 1.**

### Bibliometric analysis and software

In our study, Hist Cite, VOSviewer, CiteSpace, SCImago Graphica, Bibliometrix package and bibliometric web tool were used for analysis and visualization.

Records and total global citation score (TGCS) for each document, journal, country, producing organization, and author were calculated by Hist Cite Pro 2.1. Country collaboration map was plotted using SCImago Graphica (1.0.36) 15. Web tool (https://bibliometric.com/app) was utilized to analyze how different countries co-operated.

VOSviewer and CiteSpace are the most frequently used for bibliometric analysis 16. We used VOSviewer (1.6.19) 17 to identify high-producing institutions, journals, and authors, as well as co-cited networks. The application of Citespace V6.2 software explored the scientific trends and dynamics in this topic, as well as displayed and analyzed fresh tendency of different knowledge areas 18, and documented the clusters of co-citation references and the keywords with the strongest bursts of citations.

Bibliometrix package based on the R (version 4.2.3) and RStudio (version 2023.09.0) platforms, was used to collect comprehensive literature information, such as word clouds of keywords and three field plots.

## Results

### Annual publications trends and literature characteristics

**Figure 2:**
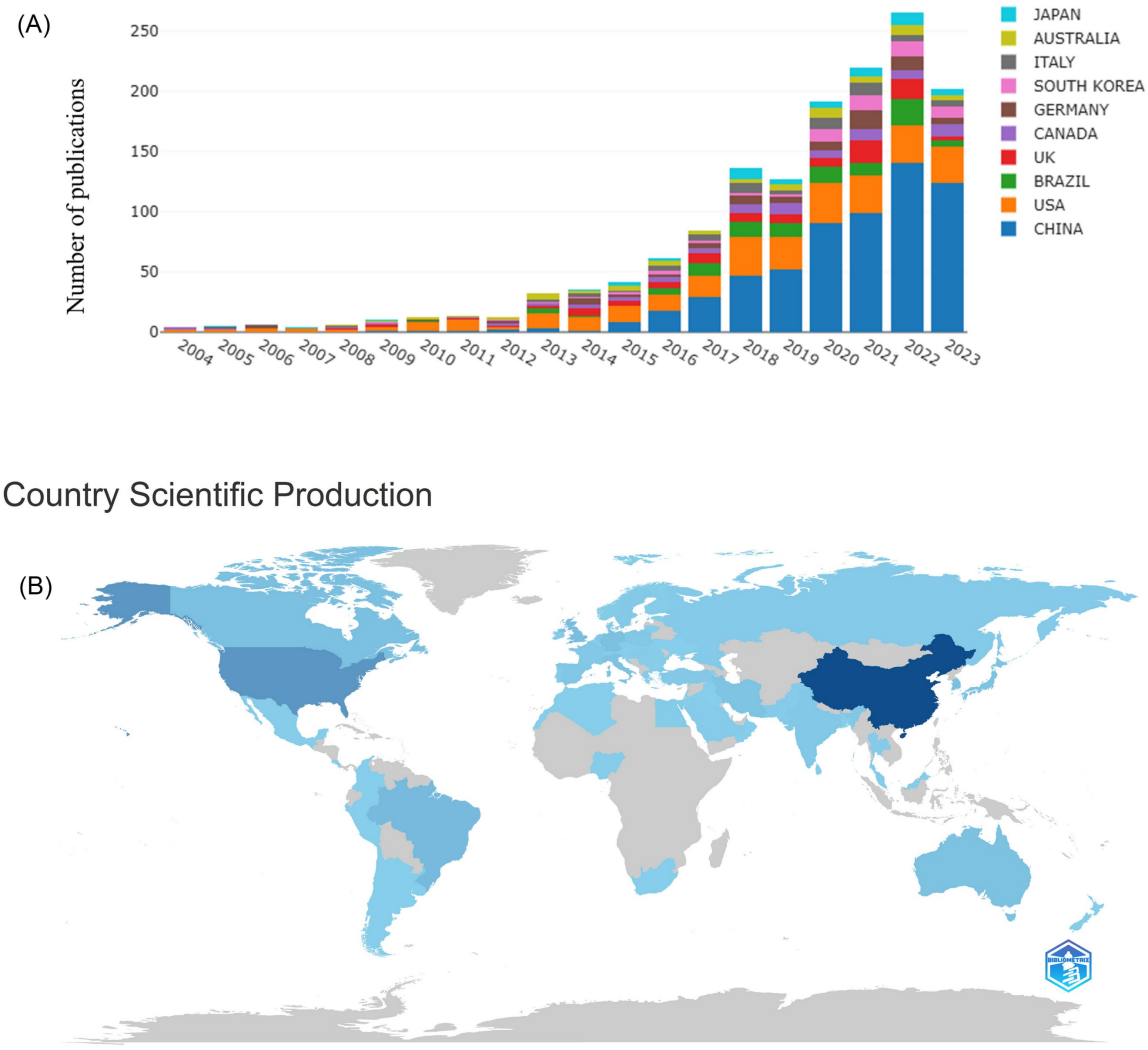
Annual output of research related to neuroinflammation in depression (A), country/region scientific production world map of neuroinflammation research in depression (B).

Over the period of 2004-2023, neuroinflammation in depression was mentioned in a total of 1,496 publications from WoSCC, with 1,205 (80.55%) articles and 291 (19.45%) reviews. Between 2004 and 2010, less than fifteen articles were produced each year on average, which revealed a modest or stagnant trend, indicating the exploration of neuroinflammation in depression appeared to be in the early stages. The number of articles rose above fifteen in 2011, and from that moment, on average, 98 articles were released annually (**Figure 2A**). The peak occurred in 2022 when 260 articles were published, with infrequent minor declines. It indicates the rapid increase of publications on neuroinflammation in depression. Although there may be intermittent obstacles, breakthroughs are still being made as new findings are made available.

### Analysis of country/region and institution characteristics

The distribution of country or region derived from the authors in the field of neuroinflammation in depression from 2004 to 2023. In **Figure 2B**, the intensity of the blue color correlates directly with the quantity of publications in the country or region. China has the most publications in this field (585), followed by the USA (285), BRAZIL (99), and UK (91). An overview of the top 10 producing countries is exhibited in **Table 1**. Additionally, in terms of top 10 cited countries, the USA ranked first, followed by China, UK, Canada, Australia, Germany, Brazil, Italy, France, and Thailand (**Table 1**).

**Figure 3:**
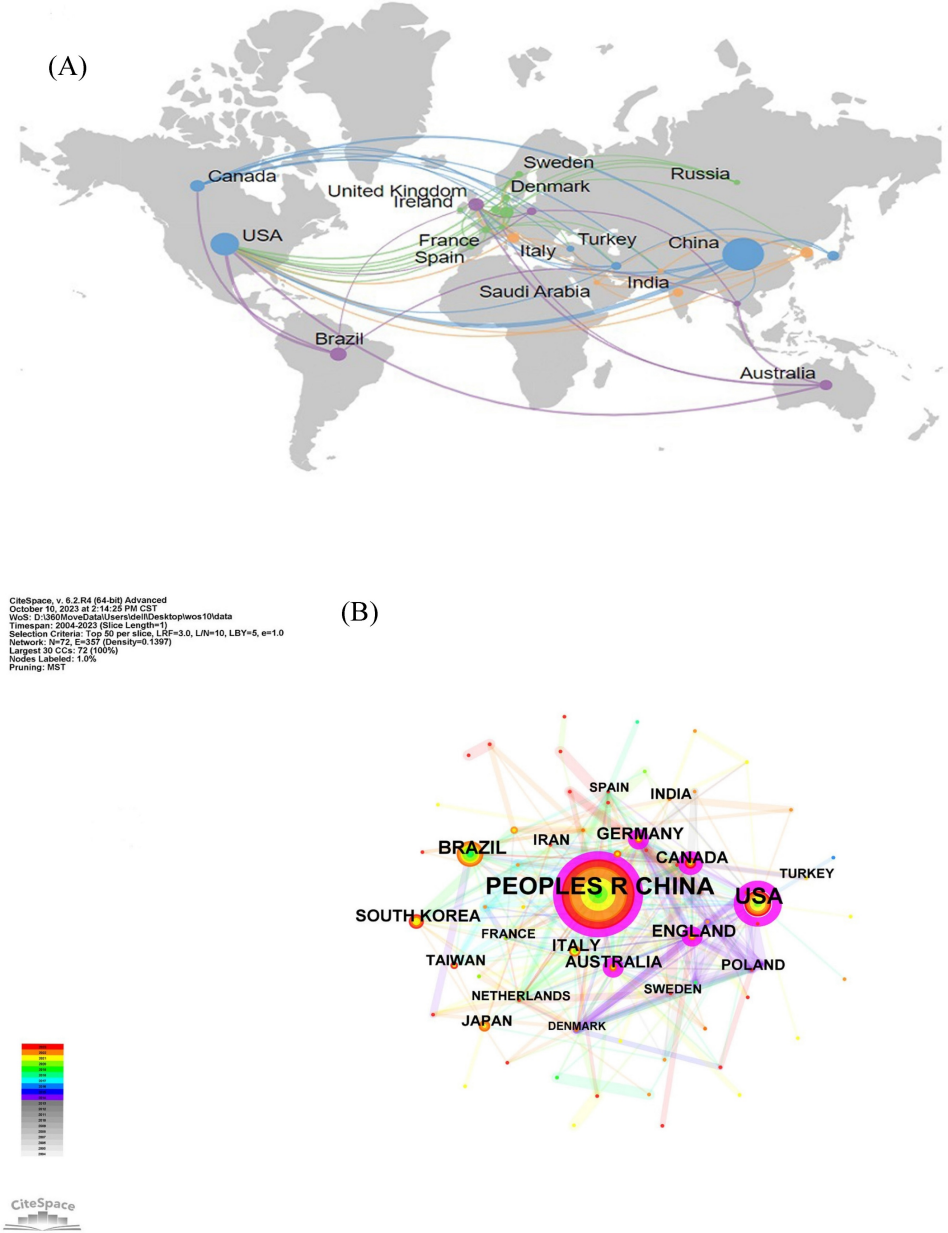

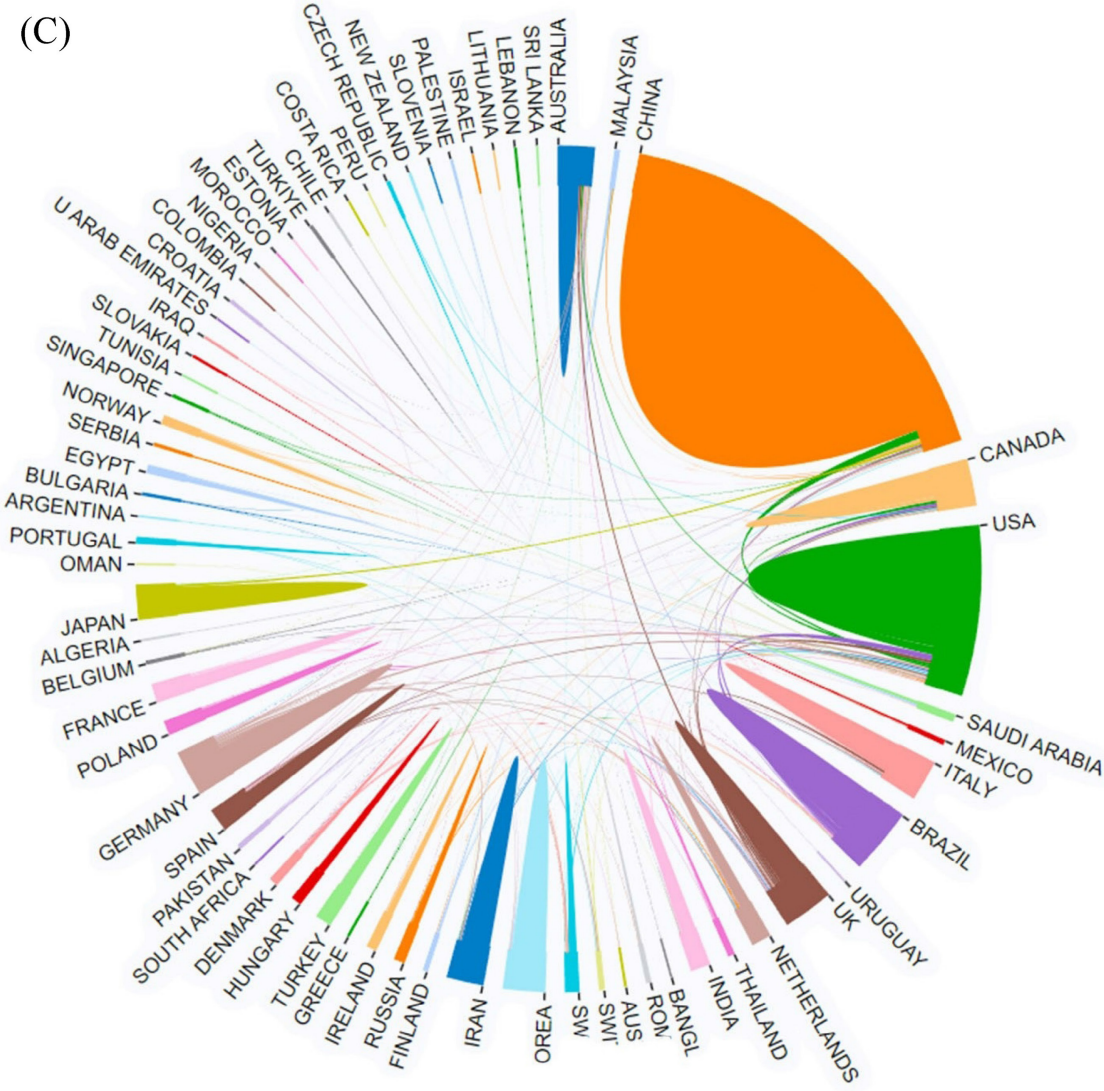
The network map of countries involved in the field of neuroinflammation in depression. (A) Country or region collaboration world map of neuroinflammation in depression. (B, C) The visualization map of collaboration among countries.

Combined with the geographical distribution analysis map drawn by SCImago graphica (**Figure 3A**), it can be seen that USA is the center of national collaborations, followed by China and Canada. A close cooperation is also taking place between European countries. In addition, the results of similar collaborations between different countries are shown in **Figure 3B** and **3C** by using CiteSpace and other online websites.

**Figure 4:**
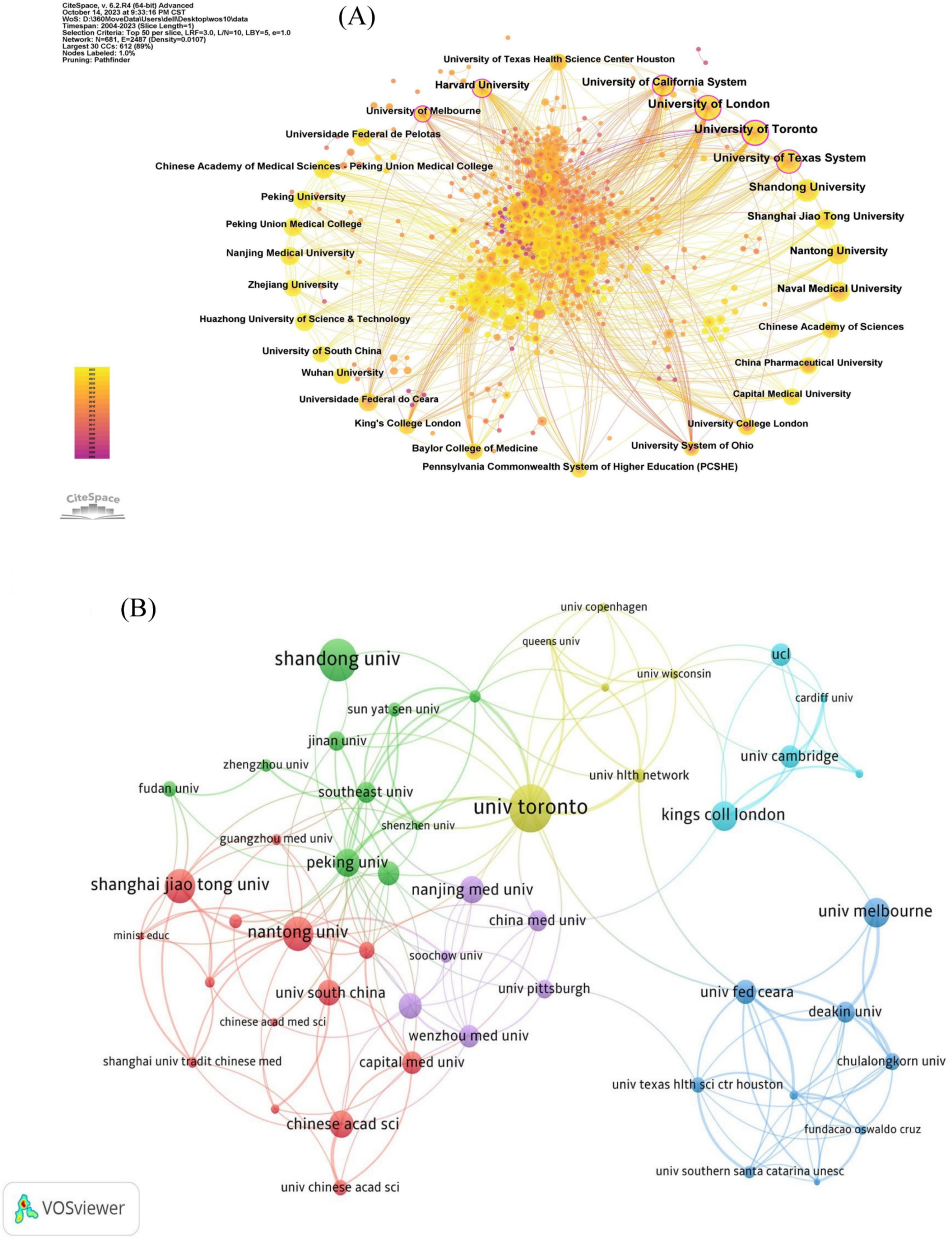

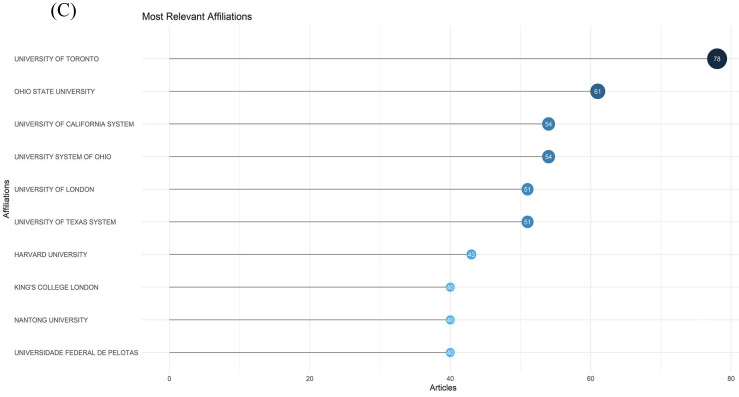
The network of institutions. (A, B) The cooperation network map of institutions. (C) Top 10 most relevant affiliations.

According to the Institutional Collaboration Network (**Figure 4A**), the links between various institutions have gradually become closer. The size of a node (and label) displays the number of published papers, where larger nodes denote a higher number of publications. The closer the year of publication is, the closer it reaches the top color of the ribbon and vice versa.

A total of 409 research institutions published articles on neuroinflammation in depression. In **Table 2**, it points the top 10 institutions with the highest publication volume in the field of study, with University Toronto ranking first with the highest number of 34 publications, indicating that its prominent influence on this research. The top five institutions in terms of average citation frequency were University Toronto (2137), University Melbourne (1069), UCL (1672), Kings Coll London (1631), and Ohio State University (1360). The close cooperation among the various agencies can be seen in **Figure 4B**. As exhibited in **Figure 4C**, the most relevant affiliations also represent their influence cannot be neglected in this research field.

### Analysis of journals

**Figure 5:**
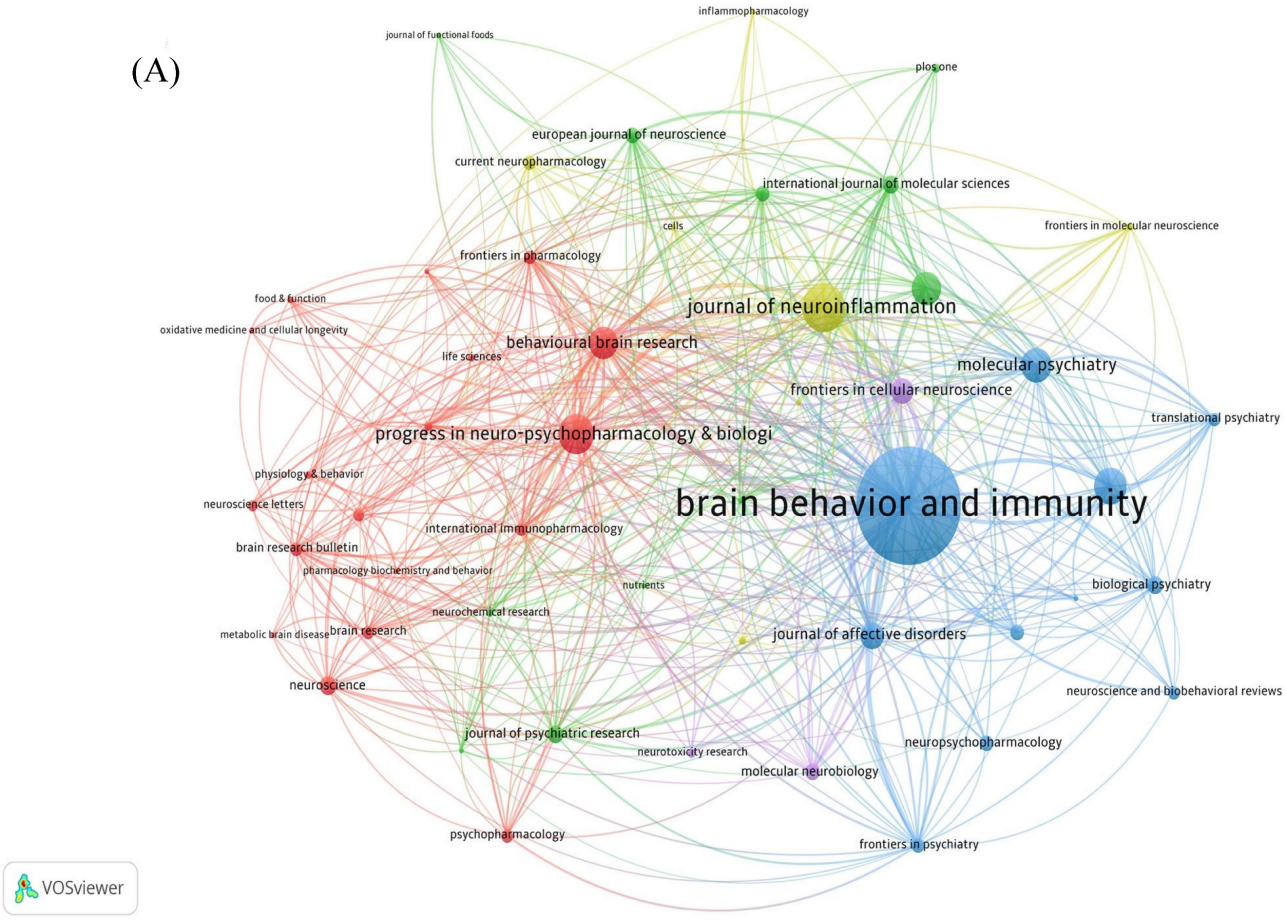

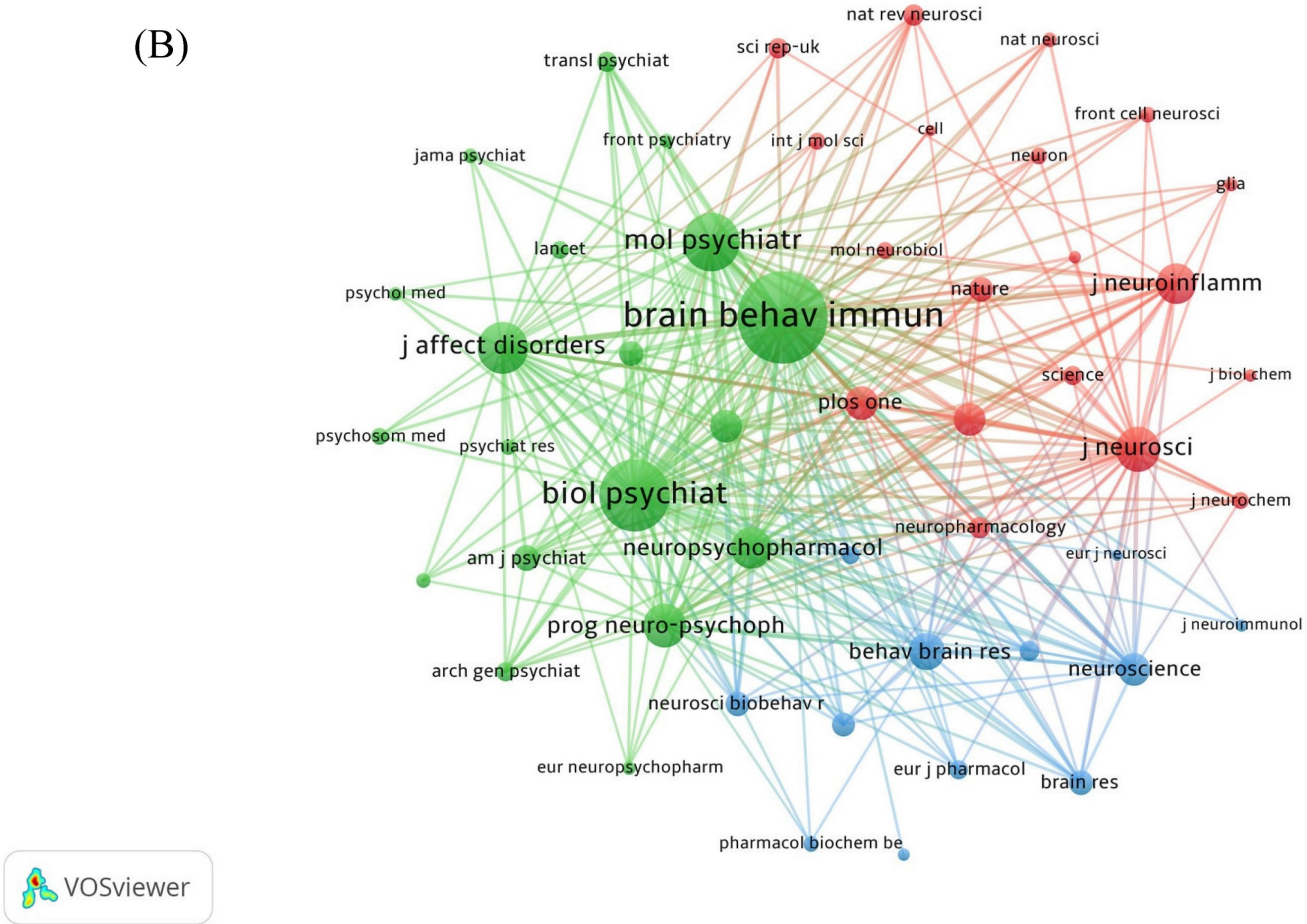

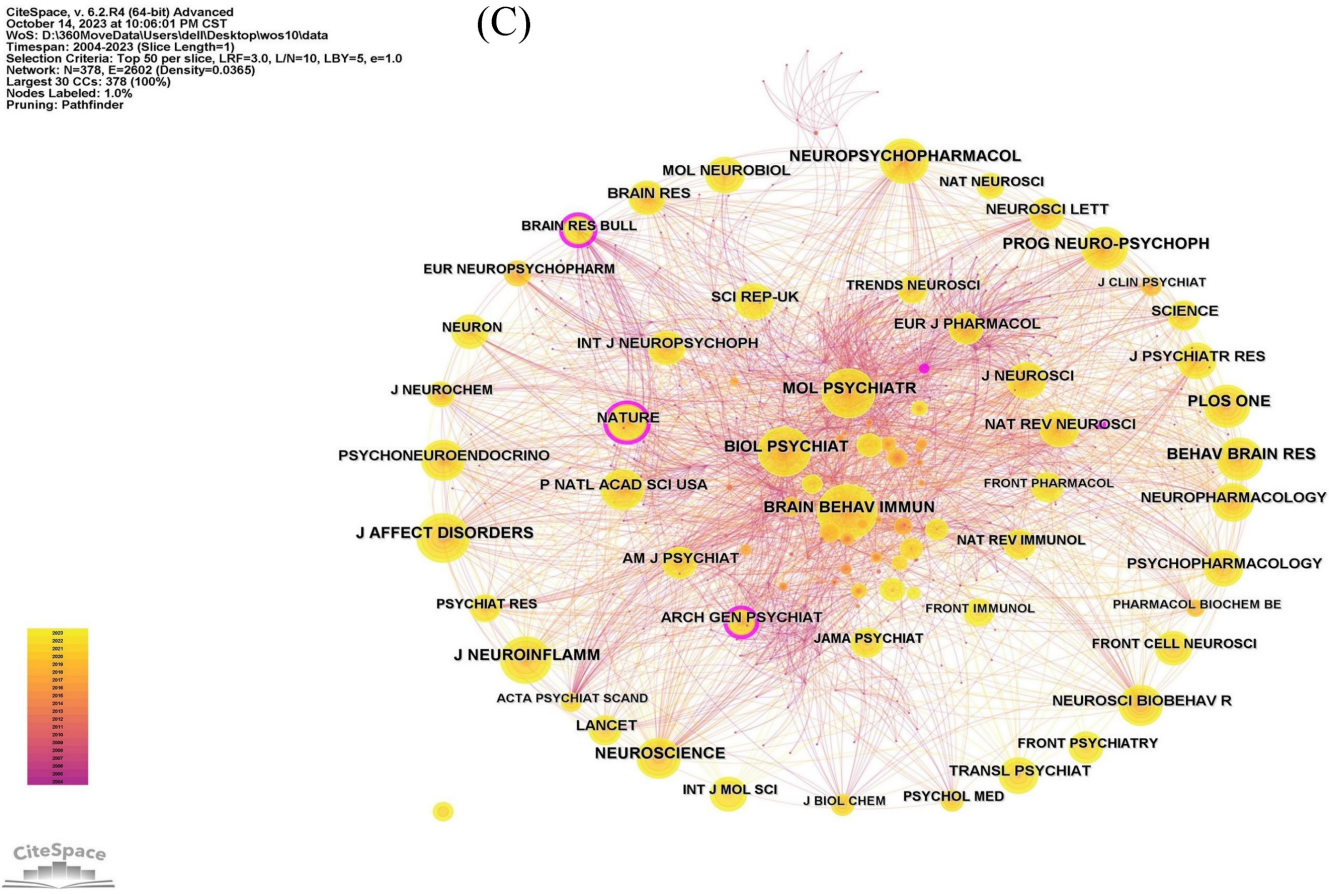
The network of journals and co-cited journals involved in the field of neuroinflammation in depression. (A) Citation analysis of journals. (B, C) Network map of co-cited journals performed by VOSviewer and CiteSpace respectively.

409 journals were involved in the publication of literature. **Table 3** indicates the top 10 journals in terms of the number of publications, the most published journal is *Brain Behavior and Immunity* with 93 articles, followed by *Journal of Affective Disorders* (53 articles) and *Behavioural Brain Research* (43 articles). Journals' cited time is closely related to its influence in the field. The network of cited journals is visualized in **Figure 5A**. The top 5 most cited journals are listed in **Table 3,** *Brain Behavior and Immunity* (TGCS=6247) ranking first with the number of 91, while the highest IF journal is *JAMA Psychiatry* (IF=25.8).

The analysis of co-cited journals contributes to identify the journals that are frequently cited and learn more quickly about journals that support the field of neuroinflammation in depression. In **Figure 5B**, the top three journals with co-citations were *Brain Behavior and Immunity* (IF=15.1004), *Biological Psychiatry* (IF=10.6002), and *Molecular Psychiatry* (IF=11.0003). The network of co-cited journals is also visualized in **Figure 5C**.

Bradford's Law is one of the three laws of bibliometrics, which is a method to sort journals and identify key journals within a particular subject area on the basis of the mathematical formula 1:n:n^2^^19^. It suggests that a small core of journals will contribute a large portion of the articles on a specific topic. According to this law, **Figure S1** shows the top 15 core journals, all of which hold significant influence in this field.

### Analysis of authors and cocited authors

Among the 1,496 included research papers, there are 7,543 authors, with 24 authors having a volume of ≥ 11 (high volume). Among them, Zhang Y and Li Y rank number one, publishing 31 related literatures (**Table 4**).

**Figure 6:**
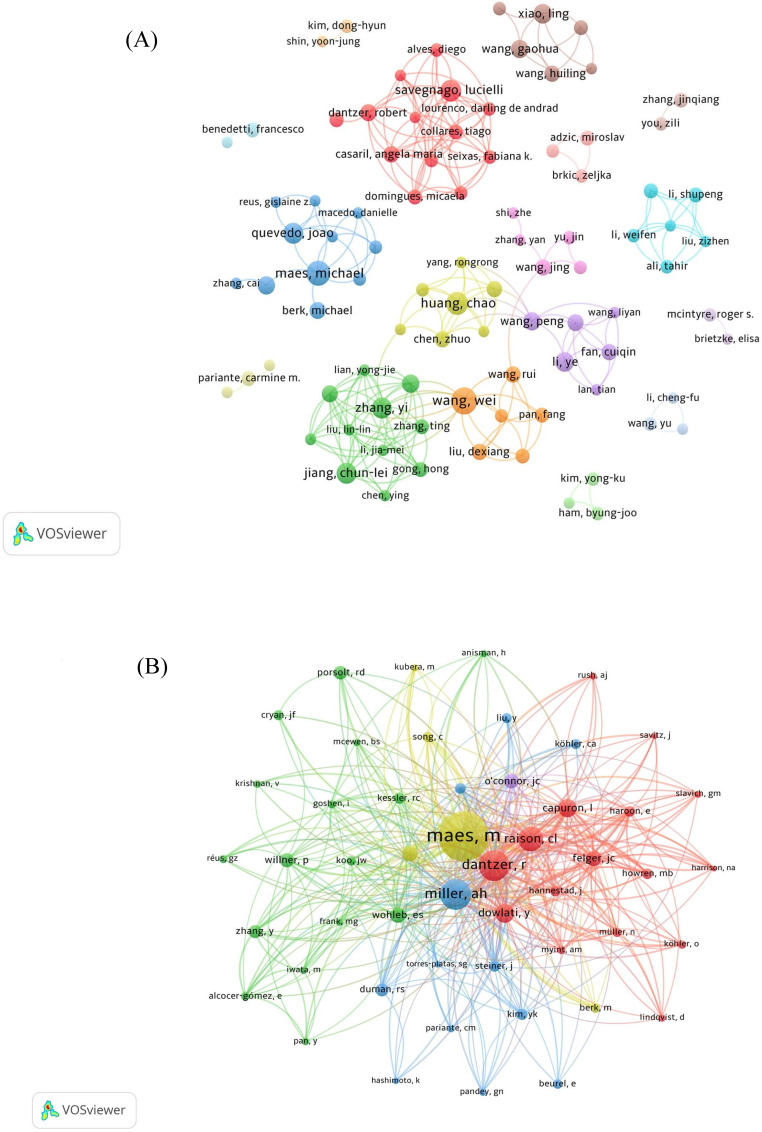

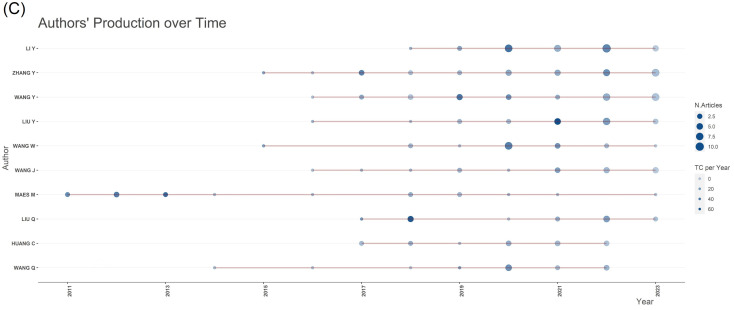
Author network analysis. (A) Collaborative web view of authors. (B) Network map of co-cited authors. (C) Top 10 authors' production over the time for neuroinflammation in depression.

A collaborative web view of authors (**Figure 6A**) shows how authors collaborate with each other and form research teams of varying sizes. The team represented by Savegnago L is larger in size and has more publications. Furthermore, there is reciprocal cooperation between the groups. However, small research groups are widely distributed, with relatively little collaboration between authors across teams. Author co-citation analysis is shown in **Figure 6B**.

As it can be seen from **Figure 6C**, Maes M, who used to produce many research outputs in this field, made a notable contribution to this field. Over the past five years, much more authors have entered this field of research, and now it is Li Y, Zhang Y, Wang Y, and Liu Y who mainly contribute to this field.

### Analysis of citation and co-cited references

**Table 5** shows the top 5 most cited articles. The top article (TGCS=734) is related to the psychopathological state of survivors after COVID-19^20^, which also taking into account the impact of inflammatory predictors in clinical experience. This article is also the latest among the top 5 most cited articles. The second most cited reference was published by Haapakoski R in* JAMA Psychiatry* (TGCS =654) 21 followed by Setiawan E (TGCS=559) 22.

**Figure 7:**
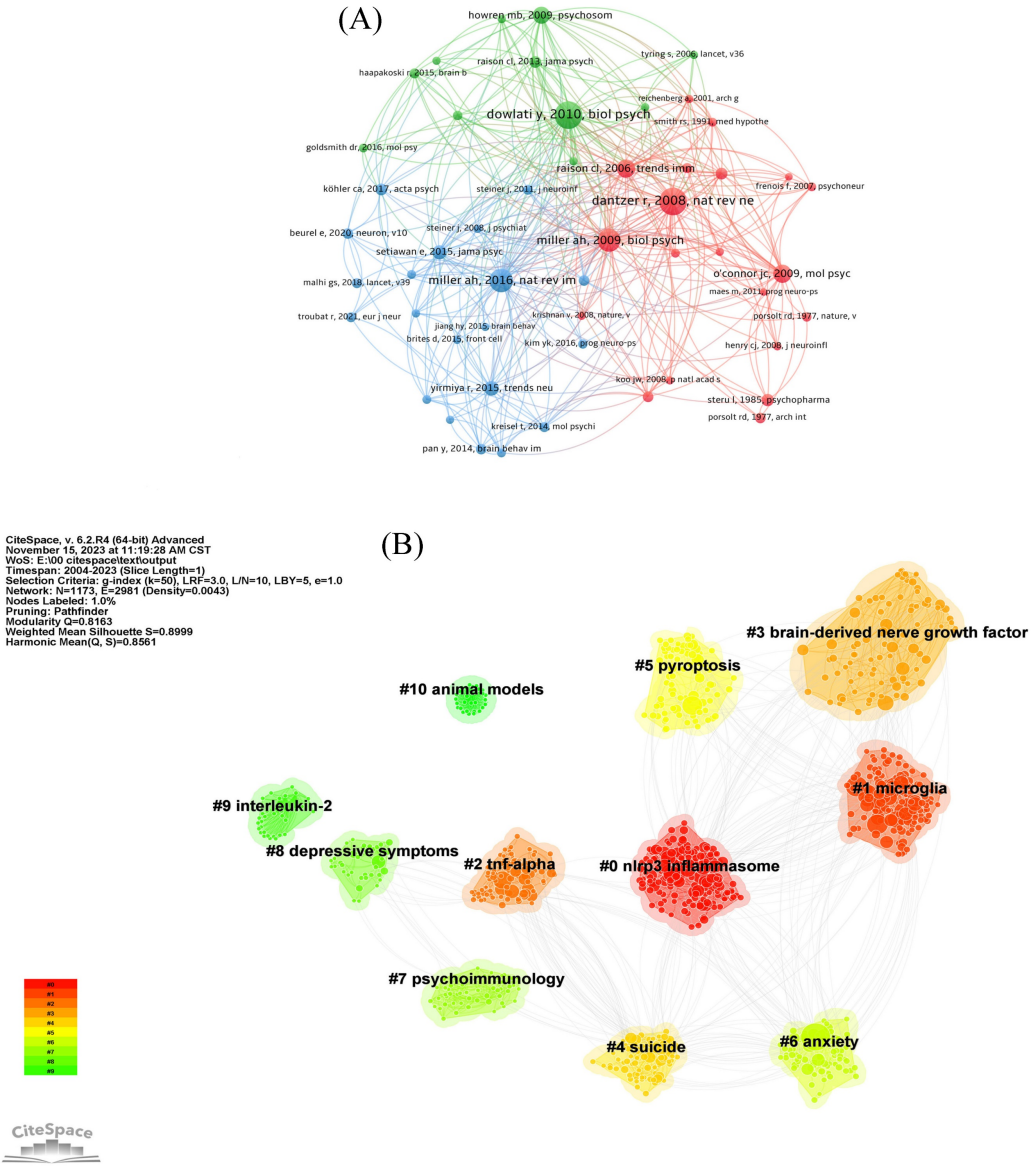
The network of references involved in the field of neuroinflammation in depression. (A) Network map of co-cited references. (B) Visualization map of major clusters of co-cited references.

The network of co-cited references is shown in **Figure 7A**. The most co-cited reference is a meta-analysis published by Dowlati Y in *Biological Psychiatry* in 2010, which suggested that depression is accompanied by IRS activation. Dantzer R published the second co-cited reference in 2008. This article suggests the stimulation of inflammation as an important biological event for the risk of occurrence of depression. The paper which was written by Miller AH and published by *Biological Psychiatry* in 2009 followed suit 25.

Co-citation reference clustering (**Figure 7B**) shows that the clustering mainly focused on the mechanism of neuroinflammation in depression (#1, #5), important factors involved in neuroinflammation hypothesis of depression (#0, #2, #3, #9), clinical symptoms associated with depression (#4, #6, #8), the subject of research (#7), and fundamental research (#10).

### Analysis of keywords

**Figure 8:**
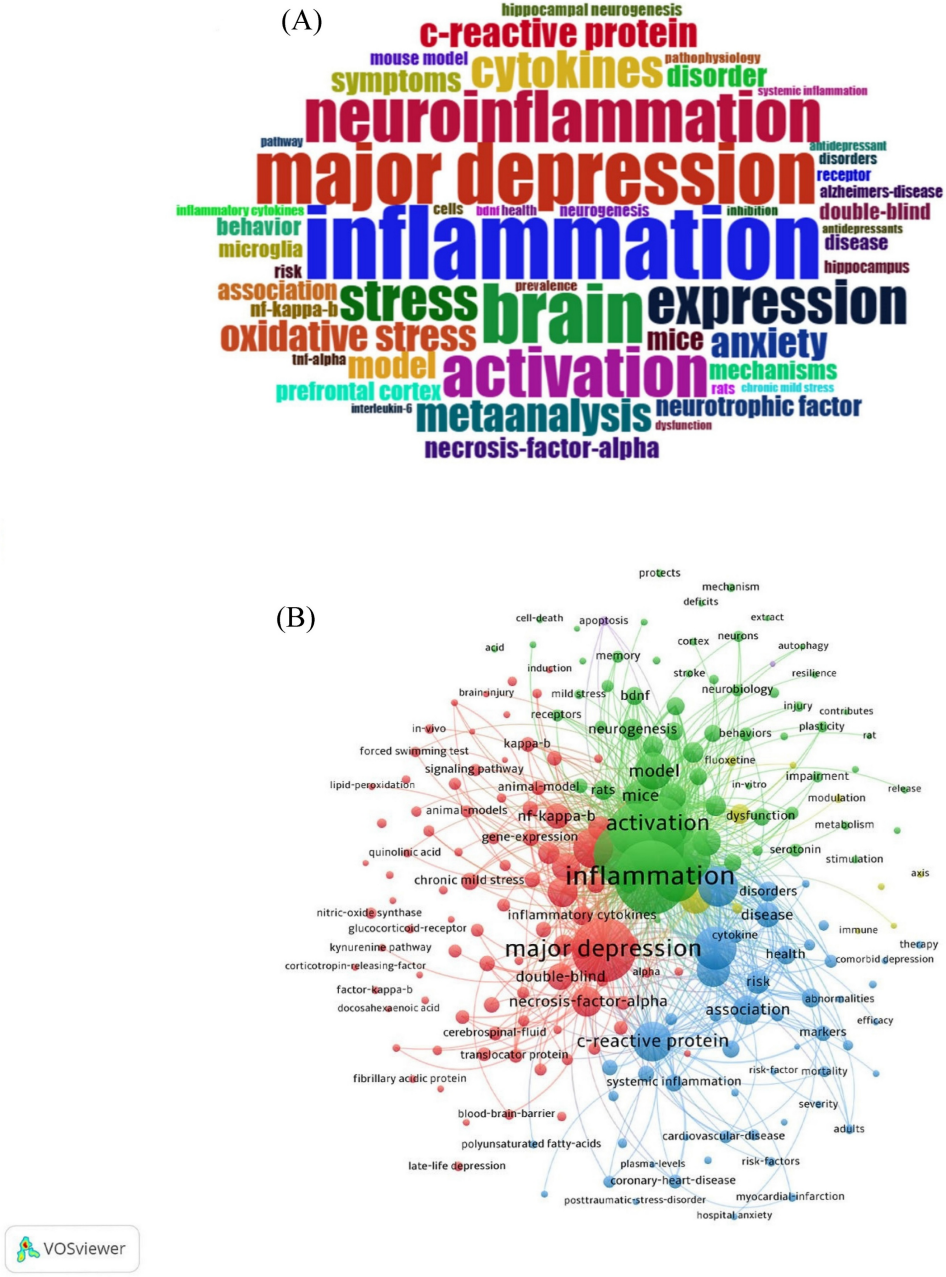
(A) Word cloud of the most frequent keywords in the neuroinflammation field in depression. (B) Cooccurrence visualization of keywords.

In order to better analyze the hot spots in this research field, 3246 keywords were ascertained, and 68 of these keywords have a frequency of more than 30. In Keywords cloud (**Figure 8A**), font size determines the frequency. The most frequency word is inflammation, major depression, brain, neuroinflammation, and activation are the next in line. The latest research focus is reflected based on the cooccurrence visualization of keywords (**Figure 8B**).

**Figure 9:**
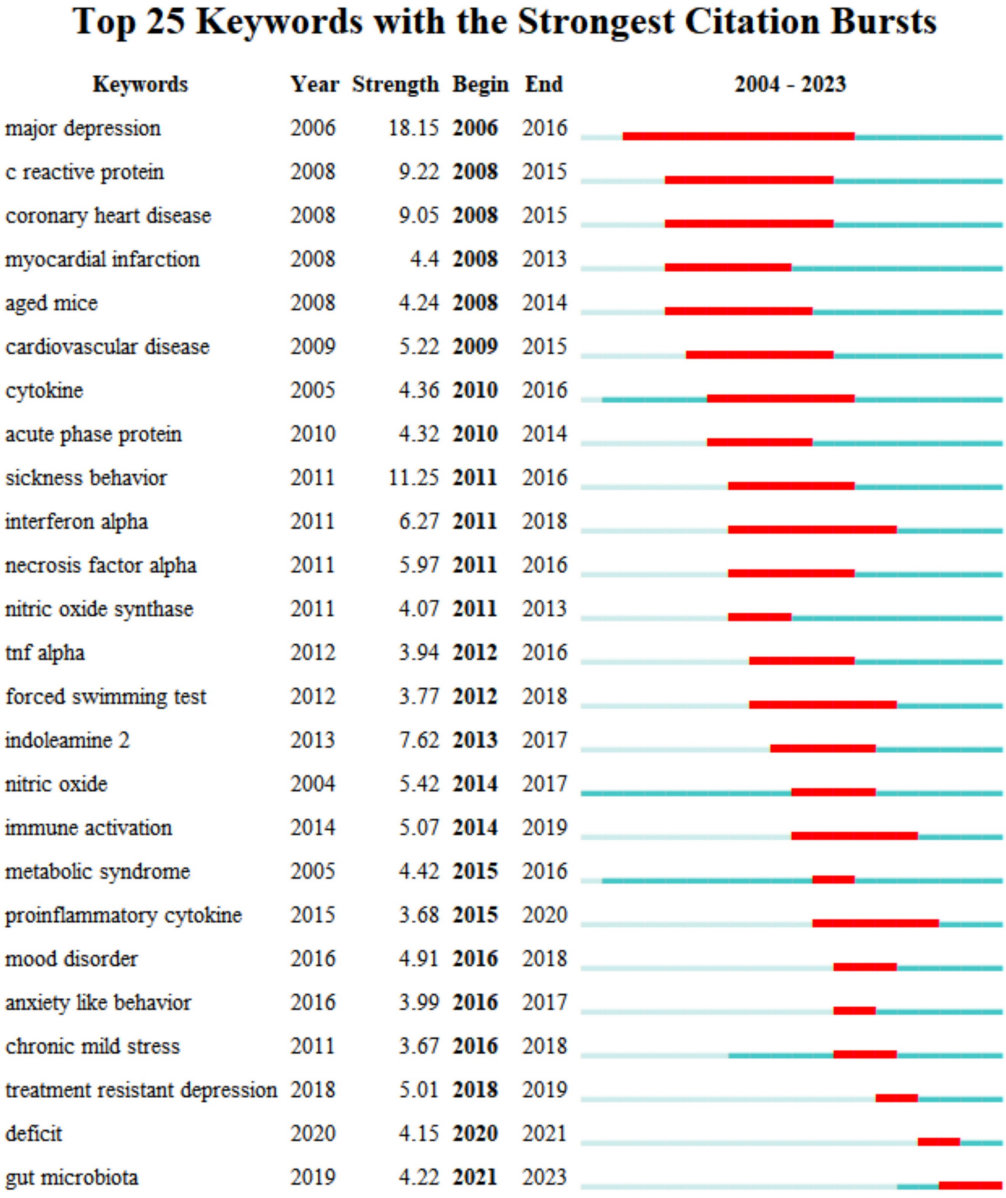
Top 25 keywords with the strongest citation burst of neuroinflammation in depression.

**Figure 10:**
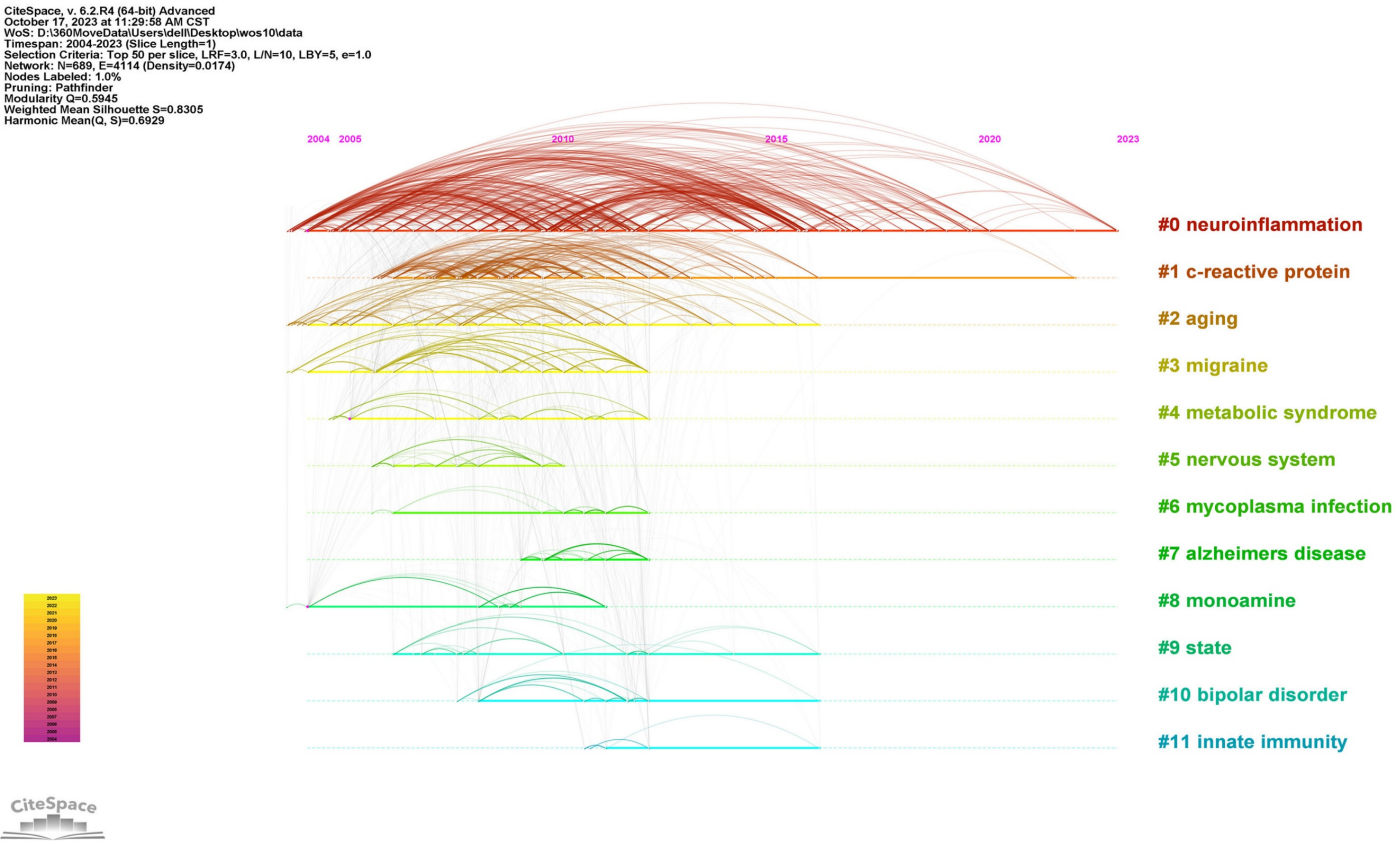
Timeline diagram in different clusters by keywords.

The top 25 keywords with the strongest citation bursts are revealed in **Figure 9**. The red line means a spike in the employment of this keyword during this period and the time range is displayed. In contrast, the blue line means insufficient popularity. In the past two decades, major depression was the first keyword burst with the strength of 18.15. Burst durations can also reveal the importance of keywords, for instance, C- reactive protein was a trending topic from 2008 to 2015, with long durations. From 2021 to the present, the newest is gut microbiota. The timeline view in different clusters by keywords is exhibited in **Figure 10**.

**Figure 11:**
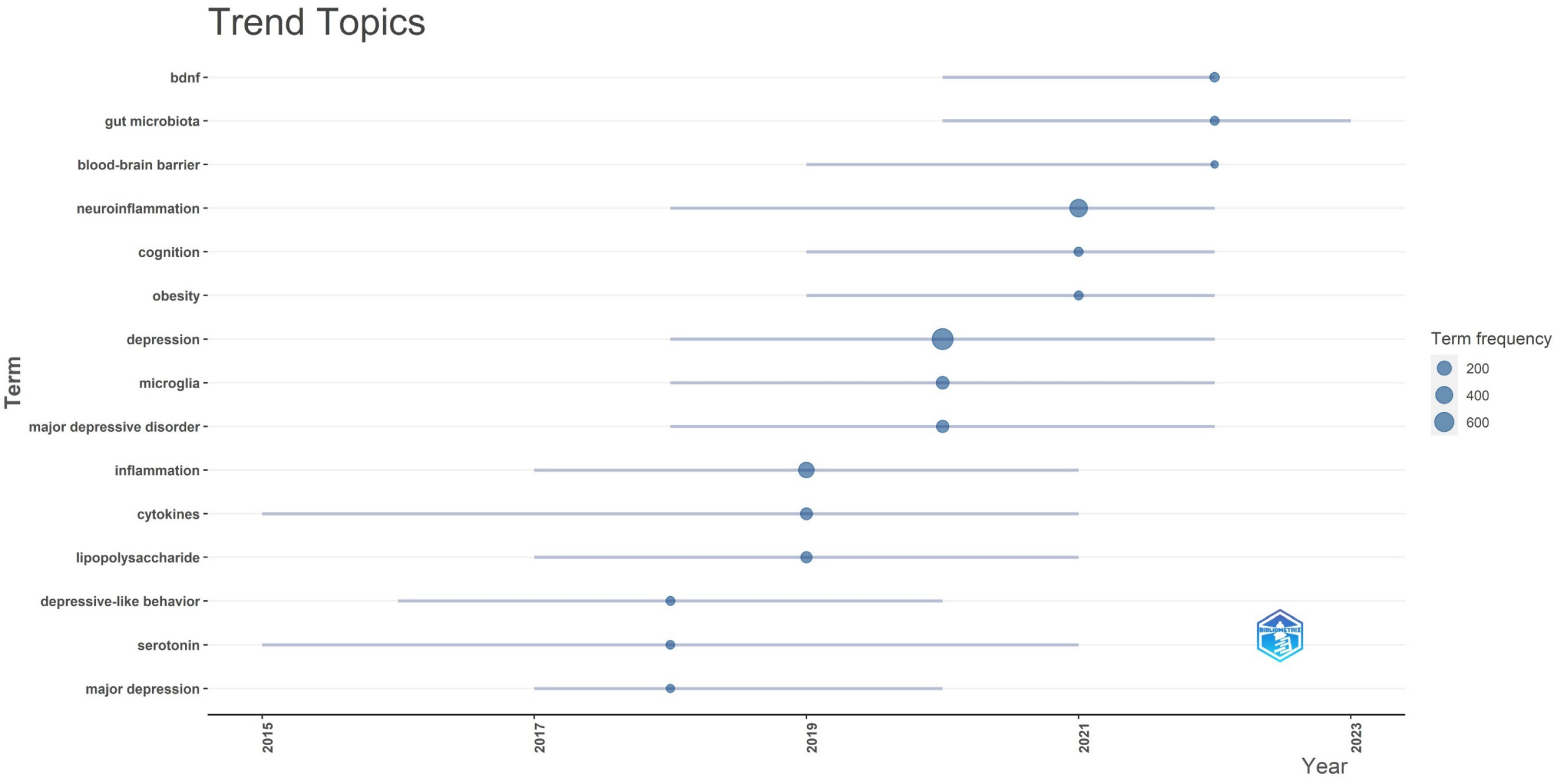
Trend topics between 2018-2023.

**Figure 12:**
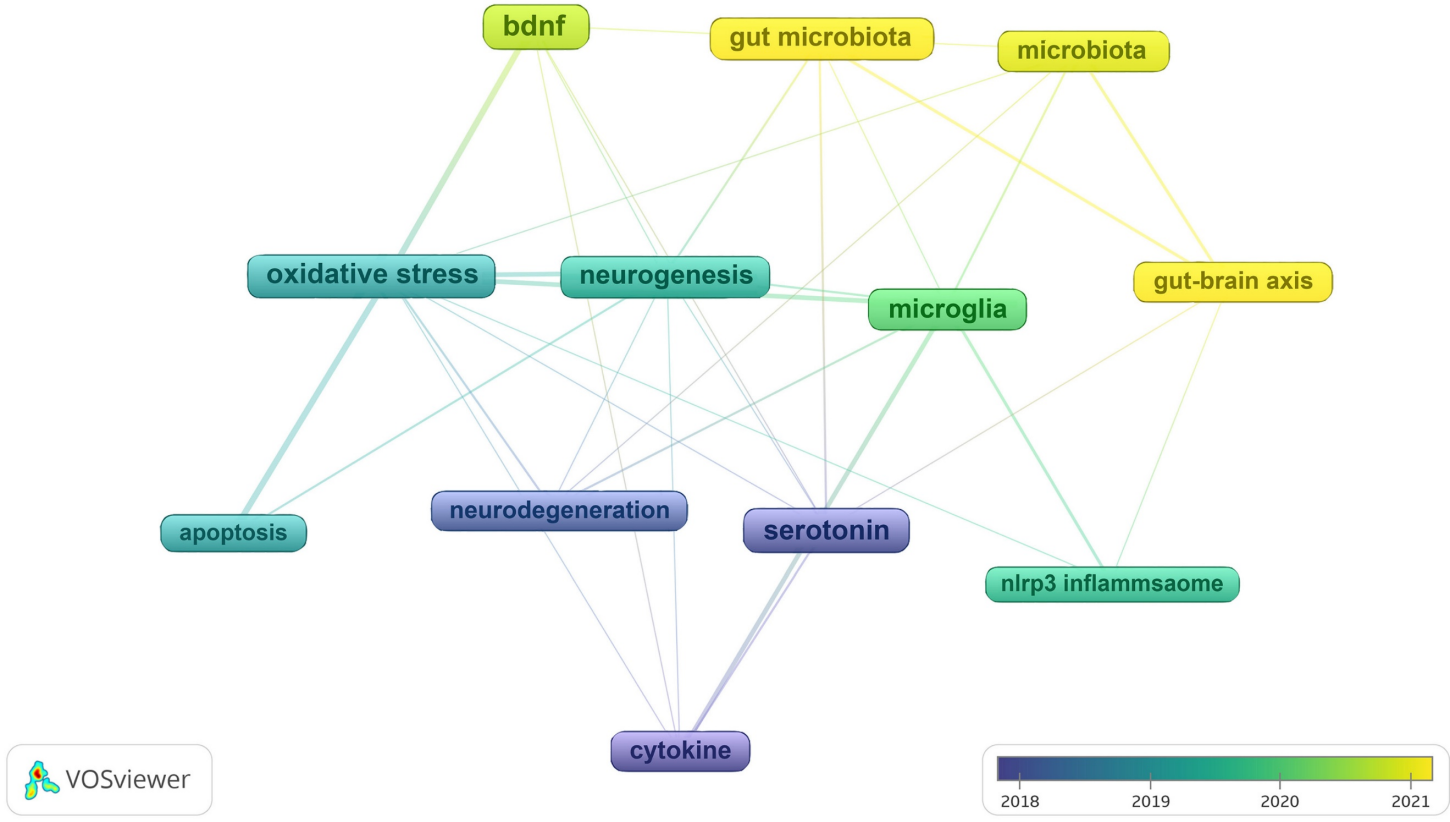
Main findings evolution.

In** Figure 11**, we analyzed trend topics in the recent five years. It can be concluded that gut microbiota dysbiosis and BDNF will be the future research trends. Along with the clustering of keywords and references, the core topics of neuroinflammation research in depression can be displayed in **Figure 12,** which includes microglia activation, the gut-brain axis, cytokine signaling, oxidative stress, neurogenesis, and so on. Among these topics, the research concerned about cytokines is the first to be studied extensively, oxidative stress and microglia were popular from 2019 to 2020, and the research on the gut-brain axis has reached its peak since 2021, and these issues are closely related.

### Relationship between authors, keywords, countries

Authors, keywords, and countries pose profound impact on a specific field. Three Fields Plot (i.e., TFP) can visualize the links among those three issues. In this plot, rectangles area correlates with the number of publications. Multiple lines connecting two rectangles represent a strong connection, as shown in **Figure S2**. Among the authors, Li Y presents the widest range of studies, which have been related to all keywords such as neuroinflammation, microglia, oxidative stress, and cytokines. Regarding countries, Chinese authors have conducted in-depth research from different perspectives with the maximum publication volume.

## Discussion

### General information

In this investigation, we investigated documents from the Web of Science database that discuss the connection between neuroinflammation and depression. A total of 1496 publications from 2004-2023 were analyzed. It is quite clear that results show an upward trend in article publication over the last 20 years. Although the collection deadline is October 2023 and there are few published documents in 2023, we could still make a conclusion that the interest in neuroinflammation mechanism of depression has increased rapidly.

In terms of country and institution-wise analysis, no more than five countries were involved in this area annually before 2008, while 72 countries have participated so far. We found that among the 72 countries involved, China and the United States have the most numerous articles and are the most cited countries, respectively. The United States has the most academic collaborations with other countries and the first-ranked TGCS. However, China had a lower influence compared to the United States. Whether analyzed from the perspective of authors or institutions being cited, Chinese authors still need to increase the international impact of their research. University of Toronto published the most documents average frequency of citations among all institutions, suggesting the most productivity and influence of University of Toronto on the topic. Many new findings are being produced by the University of Toronto's research teams. In May 2023, researchers from the University of Toronto published an article in *JAMA* 26. The paper reported that COVID-19 patients with persistent depressive symptoms and cognitive symptoms have an elevated translocator protein V-T, a marker for quantitative measurement of gliosis, in the dorsal putamen, anterior cingulate cortex, prefrontal cortex, ventral striatum and hippocampus, which provided evidence of inflammatory changes in the brain with elevated gliosis in these patients.

Maes M has been researching in this field for a long time and has the greatest influence. The most famous article which published in this research area discussed the function of proinflammatory cytokines in the etiology and pathophysiology of major depression 27, which has been cited 893 times. Recently, his latest published article showed that peripheral inflammation and insulin resistance could damage astrocytes and neuronal projections 28, disrupting mitochondrial transport and contributing to the occurrence and progress of major depressive disorder (MDD). The cooperation between transnational author teams is relatively scattered in our analysis. Consideration should be given in the future to the continuous strengthening of links between transnational authors. Sharing data and research results will help accelerate the progress of treatments for depression as well as promoting research into the pathogenesis of depression.

Among the top 10 core journals, *Brain Behavior and Immunity* (IF=15.1004), ranked relatively high in citations. It is worth mentioning that* Brain Behavior and Immunity* has the maximum amount of publications and citations. For instance, an article published in *Brain Behavior and Immunity* recently indicated that inhibition of MyD88 signaling is a promising therapeutic strategy for the treatment of stress-related psychiatric disorders 29.

The citation number of an article represents the importance of its research results. Among the highly cited articles, the paper written by Mazza MG and published by *Brain Behavior and Immunity* in 2020 ranked the first, which talked about the psychopathological influence of COVID-19 in survivors and took the effect of inflammatory predictors into account in clinical practice 20. The second most cited reference was also published in *Brain Behavior and Immunity*, which was a review by Haapakoski R published in 2015^21^. This article showed that the average levels of interleukin-6 (IL-6) and C-reactive protein in patients were increased, confirming the cross-sectional relationship between inflammation and MDD. One of the basic limitations of the neuroinflammation hypothesis was the lack of evidence of brain inflammation during MDD. Fortunately, an article written by Setiawan Ein and published by *JAMA Psychiatry* in the same year, was the first to confirm microglial activation from a large number of samples from MDD patients, providing strong evidence for the hypothesis that depression is related to microglial activation and neuroinflammation 22.

Drawing from the clustering of keywords and references, along with the burst terms analysis and the most relevant words, the current research topics and research progress on the mechanism of neuroinflammation in depression can be concluded as the following four topics: microglial activation, cytokine signaling, the gut-brain axis, and oxidative stress. Since 2017, research on cytokines has begun to increase. As time went on, the focus of research gradually shifted from cytokines to gut microbiota research. Four core findings are discussed in the following subsections, according to the order of their emerging in this research topic.

### Important cytokines involved in the neuroinflammation hypothesis of depression

Neuroinflammation is the nervous system's immune response to an inflammatory stimulus, which could be defined as an inflammatory response caused by dysregulated synthesis and release of pro- and anti-inflammatory cytokines of central or peripheral origin following damage to the organism 30. Since the 1990s, the biological relationship between immune response, neuroinflammation and depression has attracted the attention of scholars for the first time 31-33. Clinical studies have confirmed that depressed people often have an abnormal immune system and impaired immune function of peripheral cells 34. Pro-inflammatory cytokines levels, such as IL-1, IL-6, and tumor necrosis factor (TNF) -α, were significantly raised in depression patients compared with normal subjects 35,36. Pro-inflammatory cytokines are crucial in the pathogenesis of depression, and evidence indicated that pro-inflammatory cytokines could induce depression mostly by affecting monoamine neurotransmitters, the HPA axis, and reducing BDNF 37. Hippocampus, as regions with cognitive functions, has high concentrations of pro-inflammatory cytokine receptors 38. Neurogenesis has a close relationship with pro-inflammatory cytokines and neuroinflammation 39. In recent years, a growing number of studies have confirmed that pro-inflammatory cytokines lay deleterious impact on the hippocampal neurogenesis 40,41. Following the activation of microglia, increased concentrations of proinflammatory cytokines, such as IL-6 and TNF-α inhibited hippocampal neurogenesis.

Our research demonstrated that the keyword “indoleamine-2,3-dioxygenase” (IDO), which can be activated by pro-inflammatory cytokines. The activation results in the depletion of the raw material for 5-hydroxytryptamine synthesis (5-HT) 42. Some of the metabolites are also neurotoxic and these metabolites could lead to neurodegeneration. Blocking IDO can weaken the activation of microglia in prefrontal lobe and hippocampus, which can prevent depression 43. In Mingoti's research 44, it confirmed that MDD secondary to COVID-19 was related to the process in which inflammatory cytokines could activate HPA axis and the IDO enzyme in the state of high inflammation. Activation of IDO can lead to a reduction in tryptophan levels and an increase in toxic metabolites which are part of the kynurenine pathway. It is possible for this increase to cause activation of glial cells, neuroinflammation, toxicity, and ultimate neuronal death.

Cytokine-targeted therapy for depression has a certain potential. The application of anti-inflammatory cytokine medications in the management of depressive disorder may also offer promising avenues for drug development. A meta-analysis comprising 36 randomized controlled studies revealed that anti-inflammatory treatments, including non-steroidal anti-inflammatory drugs (NSAIDs), cytokine inhibitors and glucocorticoids, have a beneficial antidepressant effect in patients with depression 45. These drugs achieved the purpose by reducing the level of central inflammatory cytokines in patients with depression 46. Recently, the result of a new research 47 suggested that inflammatory cytokines could be reduced by weight-loss medications. However, due to the small sample size, and differences in depression severity, age, and disease history, these results remained a certain heterogeneity. Improvements could be targeted for further research. In summary, it suggests that researchers can develop targeted novel therapeutic strategies in depression by acquiring a more profound comprehension of the mechanisms of anti-inflammatory cytokines, and develop cytokine-based diagnosis and efficacy prediction models combining new technological tools.

### Interaction between oxidative stress and neuroinflammation in depression

In the late 1990s, research indicated a correlation between depressive symptoms and a reduction in antioxidant levels in plasma. The role of reactive oxygen species (ROS) in the pathogenesis of depression has been clarified 48. Firstly, oxidative stress can cause an inflammatory response. When cells are affected by oxidative stress, they release some signal molecules to activate the inflammatory response 49. On the other hand, microglial activation to the M1 phenotype during the neuroinflammation process and release a number of inflammatory mediators. As a result, oxidative stress injury occurs in turn harmful to the central nervous system, ultimately leading to depression 50. Previous studies have confirmed this link. For example, Baghaei Naeini F recently demonstrated that resveratrol could reduce depression-like behavior in stressed animals 51, and induce changes in anti-inflammatory and pro-inflammatory cytokines, as well as antioxidant effects including regulating superoxide dismutase and catalase activities in the hippocampus. Quercetin supplementation in mice also reduced oxidative stress and neuroinflammation induced by lipopolysaccharide (LPS) and ameliorated behavioral abnormalities 52. New research explored the association between oxidative balance score and depressive symptoms, providing a new diagnostic reference for depressive symptoms 53.

### Mechanism of microglial activation in depression

As shown in **Figure 7B**, the cluster microglia significantly involved in this topic. Back in 2018, microglia began to be popular in the field of neuroinflammation of depression (**Figure 11**). Microglia is a type of innate immune cells found in the central nervous system. During the pathogenesis of depression, microglia are polarized and differentiate into M1 microglia to trigger neuroinflammation, and M2 microglia to inhibit inflammatory factors and participate in the protection of neural tissues. Homeostasis between M1 and M2 types is essential in depression 54. Multiple signaling pathways are closely bound up with the abnormal activation of microglia, which also influence the pathological process of depression. Activated microglia release inflammatory cytokines, such as interleukin-1β (IL-1β), TNF-α, IL-6 and inducible nitric oxide synthase. Individuals suffering from depression exhibit increased levels of inflammatory markers in their hippocampus during microglia activation 55. NOD-like receptor of protein 3 (NLRP3) inflammation may be involved in LPS-induced depression-like pathological changes and promote IL-1β secretion and synthesis. Moreover, M1 microglia are likely to release reactive oxygen species and IL-1β through intracellular NLRP3 and inflammatory activation 56. Xu proved that inhibited nuclear factor kappa B (NF-κB) activation mediated by the HMGB1/TLR4/NF-κB and TNF-α/TNFR1/NF-κB pathways could attenuate microglia activation and neuroinflammation 57. Another study demonstrated that microglia activation is related to depression through JAK/STAT pathway 58.

The research on microglia activation in the field of depression has made remarkable progress in recent years. Activated microglia may regulate the neurogenesis of rat hippocampus by releasing exosomes, a new way of intercellular communication 59. Depressive symptoms are also associated with abnormal microglia autophagy 60. Such research has explored the specific role of microglia in depression more accurately and may lead to new therapeutic approaches. For example, gene editing is applied to precisely regulate microglia function in rats 61, and some traditional Chinese medicine components and natural products may inhibit microglia activation 62.

### Gut microbiota in depression

The research on intestinal flora in the field of neuroinflammation of depression has become the mainstream research content since 2021. From the keywords cloud (**Figure 8A**) and top 25 strongest citation keywords (**Figure 10**), we could conclude the long-standing and well-known evidence that depressed patients have mild chronic inflammation and chronic stress. Mental stress has a serious impact on neural nerve function in the brain, which could reduce the number of intestinal lactobacillus and increase the susceptibility to infection. The correlation between NLRP3 inflammasome and depression can also be reflected in intestinal flora 63. Immune-inflammatory reaction is the key factor of depression caused by gut microbiome dysbiosis. In addition, intestinal permeability change caused by mental stress makes intestinal bacteria metabolites more likely to enter the brain. Those metabolites could destroy microglia-neuron interaction and affect synaptic transmission in hippocampus 64.

Microbiota-based methods have become increasingly important in the pathogenesis of depression and offered a novel therapeutic target. Yang J 65 provided strong identification that MDD is featured with the disorder of intestinal bacteriophages, bacteria, and fecal metabolites, representing the overall disorder of intestinal ecology of MDD. Another meta-analysis demonstrated that reduced genera Corprococcus and Faecalibacterium were found in depressed patients, and the depressive symptoms have been improved after using probiotics in the intervention study 66. Microbial-targeted therapeutics, such as dietary intervention, fecal microbiota transplantation, and probiotics, are confirmed to have an antidepressant-like effect 67. However, gut microbiota may be influenced by many factors. How to determine the characteristic microbial species related to depression after excluding confounding factors will become a new research direction in the future. The relationship between bacteria and the interaction between bacteria and metabolites needs further study.

Elevated levels of inflammation are recognized as one of the mechanisms contributing to the onset of MDD. Inflammatory factors can not only predict the response to antidepressant treatment but also predict the efficacy of anti-inflammatory drugs in the adjuvant treatment of depression. Given the topics identified in this bibliometric analysis are being explored, new targeted therapies in the field of neuroinflammation are making more breakthroughs in the clinic.

It should be noted that not all anti-inflammatory treatments have the potential to induce antidepressant effects in patients with depressive disorders. The application of anti-inflammatory interventions is inflammatory-activity dependent. In a multicenter, randomized, double-blind placebo-controlled study 68, no difference in efficacy from placebo was found in adolescents aged 15-25 years with depression who used aspirin as adjunctive therapy. It has been proposed that not all patients with a depressive disorder will exhibit the activation of the inflammatory response. The subtype of depression may be involved in this phenomenon. Future studies should be needed to address these questions.

## Limitations

Inevitably, our study has some shortcomings. First, due to objective factors, only the data from WOSCC (SCIE) is involved, indicating the possibility of ignoring the contributions of other journals. Second, across bibliometric analysis, the language is limited to English and non-English publications are excluded, which may lead to the omission of other important non-English documents.

## Conclusions

In this study, during the past two decades, 1,496 literatures were retrieved to make clear the present situation and future trends on the topic of neuroinflammation in depression, with the help of HistCite, Bibliometrix, CiteSpace, and VOSviewer. The number of publications has increased rapidly. The leading countries are China and the USA, with many cooperations of each side. In addition, *Brain Behavior and Immunity* is the leading journal that regularly publishes research in this field. Microglial activation, gut microbiota, cytokine signaling, and oxidative stress were research hotspots in recent years. In the future, chronic stress, hippocampal structure, and gut microbiota will continue to be studied in the field of neuroinflammation in depression. This study may benefit scientists in identifying potential directions for future study, and providing clinicians with new ideas and worthy information for treatment.

## Supplementary Material

Supplementary figures.

## Figures and Tables

**Table 1:** Top 10 productive countries and most cited countries.

Rank	Most productive countries				Most cited countries			
	Country	Records	TLCS	TGCS	Country	Records	TLCS	TGCS
1	Peoples R China	585	922	11436	USA	285	963	12986
2	USA	285	963	12986	Peoples R China	585	922	11436
3	Brazil	99	300	2672	UK	91	370	5317
4	UK	91	370	5317	Canada	76	394	4084
5	Canada	76	394	4084	Australia	59	171	3548
6	Germany	69	238	2818	Germany	69	238	2818
7	South Korea	62	131	1466	Brazil	99	300	2672
8	Australia	59	171	3548	Italy	59	110	2641
9	Italy	59	110	2641	France	25	253	2512
10	Japan	51	108	1538	Thailand	18	112	1660

**Table 2:** Top 10 most productive and cited institutions involved in the field of neuroinflammation in depression.

Rank	Most productive institutions				Most cited institutions			
	Institution	Records	TLCS	TGCS	Institution	Records	TLCS	TGCS
1	Univ Toronto	34	270	2137	Univ Toronto	34	270	2137
2	Shandong Univ	30	48	642	Univ Melbourne	21	68	1698
3	Nantong Univ	24	60	490	UCL	16	115	1672
4	Shanghai Jiao Tong Univ	24	45	418	Kings Coll London	21	134	1631
5	Kings Coll London	21	134	1631	Ohio State Univ	14	101	1360
6	Univ Melbourne	21	68	1698	Univ Illinois	15	78	1323
7	Peking Univ	20	35	422	Deakin Univ	15	50	1151
8	Chinese Acad Sci	19	40	440	Second Mil Med Univ	18	108	1076
9	Nanjing Med Univ	9	34	276	Univ Cambridge	16	64	1063
10	Second Mil Med Univ	8	108	1076	Univ Vita Salute San Raffaele	7	26	1019

**Table 3:** Top 10 productive journals and top 10 most cited journals.

Rank	Most productive journals	Records	TGCS	IF(2022)	Most cited journals	Records	TGCS	IF(2022)
1	*Brain Behavior and Immunity*	93	6247	15.1004	*Brain Behavior and Immunity*	93	6247	15.1004
2	*Journal of Affective Disorders*	53	978	6.6002	*Journal of Neuroinflammation*	41	2043	9.2994
3	*Behavioural Brain Research*	43	1211	2.7001	*Progress in Neuro-Psychopharmacology & Biological psychiatry*	25	1578	5.5999
4	*Journal of Neuroinflammation*	41	2043	9.2994	*JAMA Psychiatry*	6	1424	25.8005
5	*International Journal of Molecular Sciences*	29	572	5.5999	*Molecular Psychiatry*	18	1356	11.0003
6	*Frontiers in Psychiatry*	28	438	4.7003	*Psychoneuroendocrinology*	26	1262	3.7001
7	*Frontiers in Pharmacology*	28	393	5.5999	*Behavioural Brain Research*	43	1211	2.7001
8	*International Immunopharmacology*	27	385	5.5999	*Journal of Affective Disorders*	53	978	6.6002
9	*Psychoneuroendocrinology*	26	1262	3.7001	*Frontiers in Cellular Neuroscience*	2	905	5.2999
10	*Progress in neuro-Psychopharmacology & Biological psychiatry*	25	1578	5.5999	*Neuroscience*	14	620	3.3000

**Table 4:** Top 10 productive authors and most cited authors.

Most productive authors		Most co-cited authors	
Author	Records	Co-cited author	Cited Times
Zhang Y	31	Maes M	968
Li Y	31	Miller AH	521
Wang Y	30	Dantzer R	517
Liu Y	21	Raison CL	404
Wang W	18	Dowlati Y	283
Wang J	17	Capuron I	274
Maes M	16	Wohleb ES	224
Liu Q	15	Yirmiya R	223
Wang Q	14	Felger JC	222
Huang C	14	O'Connor JC	210

**Table 5:** Top 5 most cited publications.

Title	First Authors	Journal	Citations
Anxiety and depression in COVID-19 survivors: Role of inflammatory and clinical predictors	Mazza MG (2020) 20	*Brain Behavior and Immunity*	734
Cumulative meta-analysis of interleukins 6 and 1β, tumor necrosis factor α and C-reactive protein in patients with major depressive disorder	Haapakoski R (2015) 21	*Brain Behavior and Immunity*	654
Role of translocator protein density, a marker of neuroinflammation, in the brain during major depressive episodes	Setiawan E (2015) 22	*JAMA Psychiatry*	559
Cytokine, sickness behavior, and depression	Dantzer R (2009) 23	*Immunology and Allergy Clinics in North America*	534
Association of serum interleukin 6 and C-reactive protein in childhood with depression and psychosis in young adult life	Khandaker GM (2014) 24	*JAMA Psychiatry*	503
