# Supplementary material for: A Bibliometric Analysis of Neuroinflammation in Depression from 2004 to 2023: Global Research Hotspots and Prospects
Source: Int J Med Sci. 2025 May 28;22(11):2700–20. doi: 10.7150/ijms.100888 (PMC12163427; doi:10.7150/ijms.100888)

Figure S1. Top 15 core journals by Bradford's Law.

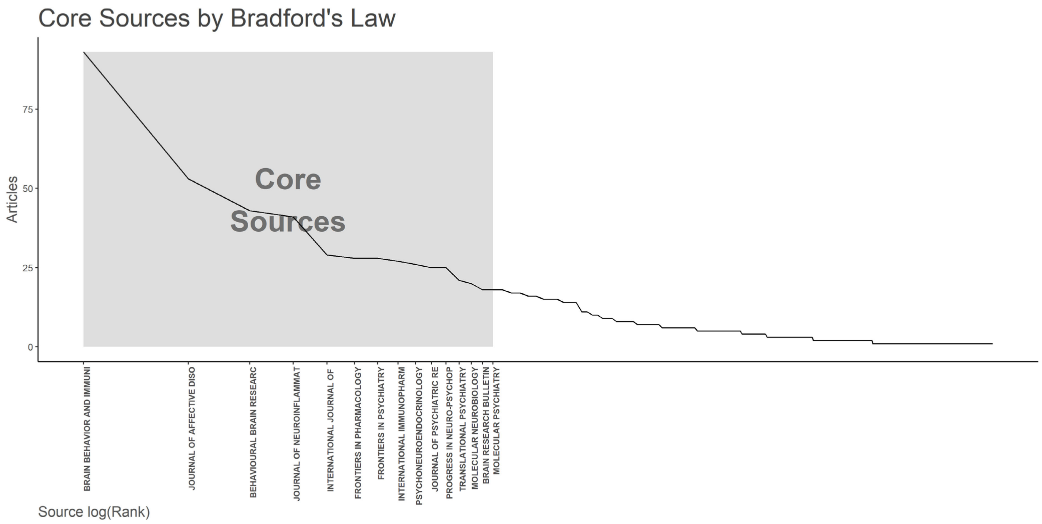

Figure S2. Relationship analysis of authors, keywords, and countries. The size of the rectangles represents the number of publications, while the connecting lines depict the correlation between academic forces, with more links indicating a higher number of studies.

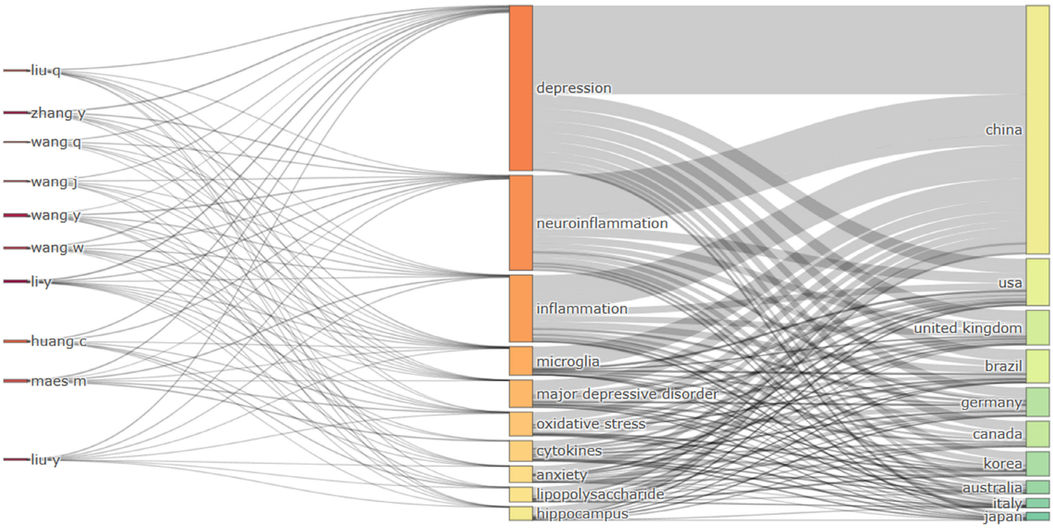

Supplement: Supplementary file 1 — Supplementary figures. [file ijmsv22p2700s1.pdf]
